# Failures of 13-Valent Conjugated Pneumococcal Vaccine in Age-Appropriately Vaccinated Children 2–59 Months of Age, Spain

**DOI:** 10.3201/eid2606.190951

**Published:** 2020-06

**Authors:** Sergi Hernández, Fernando Moraga-Llop, Alvaro Díaz, Mariona F. de Sevilla, Pilar Ciruela, Carmen Muñoz-Almagro, Gemma Codina, Magda Campins, Juan José García-García, Cristina Esteva, Conchita Izquierdo, Sebastià González-Peris, Johanna Martínez-Osorio, Sonia Uriona, Luis Salleras, Ángela Domínguez

**Affiliations:** Agència de Salut Pública de Catalunya, Generalitat de Catalunya, Barcelona, Spain (S. Hernández, P. Ciruela, C. Izquierdo);; Hospital Universitari Vall d’Hebron, Barcelona (F. Moraga-Llop, M. Campins, S. González-Peris);; Hospital de Nens, Barcelona (A. Díaz);; Hospital Sant Joan de Déu Barcelona, Barcelona (M.F. de Sevilla, C. Muñoz-Almagro, J.J. García-García, C. Esteva, J. Martínez-Osorio);; Institut de Recerca Sant Joan de Déu, Esplugues de Llobregat, Spain (M.F. de Sevilla, C. Muñoz-Almagro, J.J. García-García);; CIBER de Epidemiología y Salud Pública, Madrid, Spain (P. Ciruela, J.J. García-García, C. Esteva, L. Salleras, Á. Domínguez);; Universitat Internacional de Catalunya, Barcelona (C. Muñoz-Almagro);; Vall d’Hebron Institut de Recerca, Barcelona (M. Campins, S. Uriona);; Universitat de Barcelona, Barcelona (L. Salleras, Á. Domínguez)

**Keywords:** Streptococcus pneumoniae, vaccine failure, serotype 3, PCV13, PCR, pneumonia, bacteria, Spain, vaccines, streptococci, respiratory infections

## Abstract

Vaccination with the 13-valent conjugated pneumococcal disease (PCV13) has reduced invasive pneumococcal disease (IPD), but there have been reports of vaccine failures. We performed a prospective study in children aged 2–59 months who received diagnoses of IPD during January 2012–June 2016 in 3 pediatric hospitals in Catalonia, Spain, a region with a PCV13 vaccination coverage of 63%. We analyzed patients who had been age-appropriately vaccinated but who developed IPD caused by PCV13 serotypes. We detected 24 vaccine failure cases. The serotypes involved were 3 (16 cases); 19A (5 cases); and 1, 6B, and 14 (1 case each). Cases were associated with children without underlying conditions, with complicated pneumonia (OR 6.65, 95% CI 1.91–23.21), and with diagnosis by PCR (OR 5.18, 95% CI 1.84–14.59). Vaccination coverage should be increased to reduce the circulation of vaccine serotypes. Continuous surveillance of cases of IPD using both culture and PCR to characterize vaccine failures is necessary.

Conjugated pneumococcal vaccines have been shown to be effective in preventing invasive pneumococcal disease (IPD) in children. In February 2001, the European Medicines Agency (EMA) authorized the use of the 7-valent pneumococcal conjugate vaccine (PCV7), which included antigens of serotypes 4, 6B, 9V, 14, 18C, 19F, and 23F (*1*) in children <2 years of age. The introduction of PCV7 led to a reduction in cases of IPD and in hospitalizations caused by vaccine serotypes (*2*,*3*). Cases of IPD with PCV7 vaccine failure represented ≈2% of all reported IPD cases and resulted mainly from bacteremia caused by serotypes 4, 6B, and 19F. Half the patients with vaccine failure had underlying diseases (*4*). In subsequent years, there was an increase in the number of IPD cases caused by non-PCV7 serotypes, mainly serotypes 1, 3, and 19A; pneumonia complicated by empyema, pleural effusion, or both was the most frequent presentation (*5*,*6*).

In April 2009 (*7*), and subsequently in December 2009 (*8*), the European Medicines Agency authorized the marketing of the 10-valent pneumococcal conjugate vaccine (PCV10), which included PCV7 serotypes plus serotypes 1, 5, and 7F. It also authorized the 13-valent conjugated pneumococcal vaccine (PCV13), which included PCV10 serotypes plus serotypes 3, 6A, and 19A. In 2010, the Vaccines Advisory Committee of the Spanish Association of Pediatrics recommended that children should receive PCV13, although the vaccine was not included in the recommended schedule of the National Health System and, therefore, parents had to pay for it. In Catalonia, Spain, PCV13 was included in the vaccination calendar financed by the public health system in July 2016. During 2012–2016, when this study was conducted, the estimated PCV13 coverage in children 7–59 months of age in Catalonia was 63% (*9*).

The introduction of PCV13 in children <5 years of age was associated with a decrease in IPD incidence caused by all vaccine serotypes, except for serotype 3, for which the decrease was not significant (*10*). Previous studies have shown that the immune response against serotype 3 after PCV13 vaccination is lower than that generated against other serotypes (*11*,*12*). Some case-control and cohort studies (*9*,*13*,*14*) have reported no effectiveness, whereas other studies have found the vaccine to be effective (*15*,*16*). Kaplan et al. (*17*) found that most cases of IPD caused by PCV13 serotypes in children receiving >1 dose of PCV13 were associated with serotype 19A and generally occurred in children <6 months of age or those with underlying conditions. However, studies have shown several cases of pneumonia complicated with empyema, pleural effusion, or both produced by serotype 3 in children vaccinated with PCV13 (*18*,*19*). We conducted a study to analyze the epidemiologic, clinical, and microbiological characteristics of cases of IPD arising from vaccine failure in children 2–59 months of age who were treated at 3 pediatric hospitals in Catalonia.

## Methods

### Study Design

We conducted a prospective study in children 2–59 months of age who received diagnoses of IPD during January 1, 2012–June 30, 2016 and were treated in 3 pediatric hospitals in Catalonia: Hospital Sant Joan de Déu, Hospital Maternoinfantil Vall d’Hebron, and Hospital de Nens de Barcelona. We estimated a reference population of 116,279 children <5 years of age (30.4% of the total population of this age in Catalonia) for the 3 hospitals (*9*).

We defined IPD as clinical findings of infection together with the isolation or detection of DNA from *Streptococcus pneumoniae* by real-time PCR in a normally sterile sample. We established the presence of *S. pneumoniae* DNA by amplification of the autolysin (*lytA*) gene and the *wzg* (*cpsA*) gene according to published assays (*20**–**22*). We included in the study only samples that were positive for the *lytA* and *wzg* genes in real-time PCR.

### Vaccination Status

We obtained the vaccination status of each case-patient from the vaccination card and the medical record of the primary care center or private center where the child was usually seen. We collected information on the number of PCV13 doses administered and the date of administration. We considered a dose valid if administered after 6 weeks of age, with a minimum interval between doses appropriate to the summary of product characteristics (SmPC) (*8*) and >15 days before the onset of IPD.

Patients were considered age-appropriately vaccinated when they had received all doses of PCV13 corresponding to their age (even when the vaccination schedule was incomplete), according to SmPC (*8*) and the Spanish Association of Pediatrics recommendations (*23*). We considered patients age-incorrectly vaccinated when they had received >1 doses of PCV13 but did not comply with any vaccination schedule included in the SmPC or had received fewer doses than they should have at disease onset.

### Identification, Serotyping, and Classification of *S. pneumoniae*

Strains of *S. pneumoniae* isolated by culture were serotyped, using the Quellung reaction or dot blot, by the National Center for Microbiology, Majadahonda, Madrid (*24*). We performed capsular typing of all culture-negative and PCR-positive samples using 2 methods, depending on the amount of *S. pneumoniae* DNA available. If the amount was low (detection of *LytA* gene DNA and *wzg* [*cpsA*] gene of *S. pneumoniae* by real-time PCR with the cycle threshold [Ct] >30 cycles), we used a previously described real-time multiplex PCR technique that detects all pneumococcal capsular types and differentiates serotypes 1, 3, 4, 5, 6A/C, 6B/D, 7F/A, 8, 9V/A/N/L, 14, 15B/C, 18C/B, 19A, 19F/B/C, 23A and 23F (*20*). If the amount of *S. pneumoniae* DNA was high (PCR-positive samples with Ct <30 cycles), we used sequential multiplex PCR combined with fragment analysis and automated fluorescent capillary electrophoresis to differentiate serotypes: 1, 2, 3, 4, 5, 6A/6B, 6C, 6,7C/(7B/40), 7F/7A, 9N/9L, 9V/9A, 10A, 10F/(10C/33C), 11A/11D, 12F/(12A/44/46), 13, 16F, 17F, 18/(18A/18B/18C/18F), 19A, 19F, 20(20A/20B), 21, 22F/22A), 23A, 23B, 24/(24A/24B/24F), 31, 34, 35A/(35C/42), 35B, 35F/47F, 38/25F, 39 (*25*). Because this procedure does not differentiate between serotypes 6A and 6C, and serotypes 7F and 7A, in 2 of the cases recorded, these serotypes were considered as nonvaccine serotypes and classified as 6A/6C and 7F/7A.

### Definition of Vaccine Failure and Demographic, Clinical, and Epidemiologic Variables

We used the definition of PCV13 vaccine failure as described by Heininger et al. (*26*). This definition is the occurrence of IPD caused by a specific vaccine-preventable serotype in a person appropriately and fully vaccinated, taking into account the incubation period and the normal delay for protection to be acquired as a result of immunization.

The following demographic, clinical, and epidemiologic variables were recorded for each case-patient: age, sex, date of birth, date of symptom onset, hospitalization date, clinical form of IPD (meningitis, septic shock, uncomplicated pneumonia, complicated pneumonia, occult bacteremia and others), complications during admission, admission to the intensive care unit (ICU), and length of stay. We also recorded medical risk conditions (sickle cell anemia, congenital or acquired asplenia, human immunodeficiency virus infection, cochlear implant, congenital immunodeficiency, chronic heart disease, chronic lung diseases including asthma if treated with a risk dose of oral corticosteroids, cerebrospinal fluid fistula, chronic renal failure including nephrotic syndrome, immunosuppressive treatment or radiotherapy, solid organ or hematopoietic progenitor transplantation, and diabetes mellitus), and date and clinical outcome at discharge (discharge without sequelae, sequelae after 6 months, death).

### Statistical Analysis

We compared categorical variables using a Pearson χ^2^ test or Fisher exact test and continuous variables using a Student *t* test. In cases of vaccine failure, we calculated the association of serotypes with age group and vaccination status for each specific serotype compared with the remaining serotypes. Values ​​of p<0.05 were considered statistically significant. We assumed a bilateral distribution for all p values​. We then calculated the odds ratios (ORs) and 95% CIs. We conducted the analyses using SPSS Statistics 19.0 (IBM, https://www.ibm.com).

### Data Confidentiality and Ethics Aspects

No diagnostic tests were made or samples taken from any participant in addition to those required by routine care. This study complies with the principles of the Declaration of Helsinki and the legal structure according to international human rights and biomedicine and personal data protection legislation. The Ethics Committee of Hospital Sant Joan de Déu approved the study. Informed consent signed by parents or legal guardians was given for all participants. All data were treated as confidential, and records were accessed anonymously.

## Results

During the study period, we recruited 188 patients 2–59 months of age who were admitted to the 3 participating centers with IPD. We identified the *S. pneumoniae* serotype causing IPD in 180 cases (95.7%), of which 104 (57.8%) were caused by PCV13 serotypes. Serotype 3 was the most frequent serotype (42 cases, 23.3%), followed by serotype 1 (19 cases, 10.6%), 19A (17 cases, 9.4%), and 14 (13 cases, 7.2%). Of the 180 case-patients, 102 (56.7%) were not vaccinated, 66 (36.6%) were age-appropriately vaccinated, and 12 (6.7%) were age-incorrectly vaccinated.

### Characteristics of Cases of Vaccine Failure

We detected 24 cases of vaccine failure according to the study definition, representing 13.3% of all cases. The serotypes identified were serotype 3 (16 cases, 66.7%); serotype 19A (5 cases, 20.8%); and serotypes 1, 14, and 6B (1 case each). The overall mean age of the 24 case-patients was 31.3 months (range 9–58 months, SD 14.7). Of the 24 case-patients, 66.7% (16) were male, and 62.6% (15) were 24–59 months of age (Table 1). The main age group was 24–59 months in cases caused by serotype 3 (81.3% vs. 25.0%; p = 0.013) and <24 months in cases caused by serotype 19A (80.0% vs. 26.3%; p = 0.047). Of the 24 case-patients, 17 (70.8%) had completed the vaccination schedule, 11 (45.8%) had a 3 + 1 dose schedule, 4 (16.7%) a 2-dose schedule, and 2 (8.3%) a 1-dose schedule. Of the 7 (29.2%) age-appropriately vaccinated patients with an incomplete vaccination schedule, 4 (16.7%) had a 3 + 0 schedule, 2 (8.3%) 1 dose, and 1 (4.2%) 2 doses. Serotype 3 was loosely associated with vaccine failure in age-appropriately vaccinated patients who had completed their vaccination schedule (OR 45.0, 95% CI 3.41–594.12). Serotype 19A was loosely associated with vaccine failure in age-appropriately vaccinated patients with an incomplete vaccination schedule (OR 21.33, 95% CI 1.75–263.67). The most frequent clinical presentation was complicated pneumonia (21/24 cases, 87.5%) and the most frequent complication of these 21 cases was empyema (15/21 cases, 71.4%). Of the 24 cases, 79.2% (19 cases) were diagnosed by PCR alone, 12.5% (3 cases) by culture alone, and 8.3% (2 cases) by PCR and culture. No patient with vaccine failure had underlying conditions.

**Table 1:** Characteristics of age-appropriately vaccinated patients 2–59 months of age with invasive pneumococcal disease caused by PCV13 serotypes, Catalonia, Spain, 2012–2016*

Pt no.	Age, mo/sex	Year	Age at vaccination	Schedule	Clinical manifestation	Complications	Outcome	DT	Ser	Vaccine schedule
1	19/F	2012	12 mo	1	Complicated pneumonia	Pleural effusion	Cured	PCR	19A	UC
2	15/M	2012	3 ,5 mo†	2 + 0	Complicated pneumonia	Empyema, pneumothorax	Cured	Culture, PCR	19A	UC
3	15/F	2012	2, 5, 8 mo	3 + 0	Complicated pneumonia	Empyema, necrotizing pneumonia, bronchoalveolar fistula, pneumothorax	Bronchopleural fistula	PCR	3	UC
4	23/M	2012	17 mo	1	Uncomplicated pneumonia		Cured	Culture	1	UC
5	24/M	2012	2, 4, 6, 18 mo	3 + 1	Complicated pneumonia	Empyema	Cured	PCR	19A	C
6	24/M	2012	12, 21 mo	2	Complicated pneumonia	Empyema	Cured	PCR	3	C
7	21/M	2012	3, 5, 7, 15 mo	3 + 1	Complicated pneumonia	Empyema	Cured	PCR	3	C
8	38/M	2012	30 mo	1	Bacteraemic mastoiditis	Epidural abscess, sigmoid sinus thrombosis	Hydrocephalus	PCR	3	C
9	9/F	2013	2, 3, 4 mo	3 + 0	Complicated pneumonia	Necrotizing pneumonia	Cured	PCR	19A	UC
10	12/M	2013	2, 4, 6 mo	3 + 0	Complicated pneumonia	Empyema, necrotizing pneumonia	Cured	PCR	6B	UC
11	50/F	2013	25 mo	1	Complicated pneumonia	Empyema	Cured	PCR	3	C
12	34/M	2013	3, 6, 9, 15 mo	3 + 1	Complicated pneumonia	Empyema, pneumothorax, bronchopleural fistula	Cured	Culture, PCR	3	C
13	15/M	2014	2, 4, 7 mo	3 + 0	Osteoarticular infection		Cured	Culture	19A	UC
14	43/M	2014	12, 15 mo	2	Complicated pneumonia	Empyema, necrotizing pneumonia, bronchopleural fistula	Cured	PCR	3	C
15	44/F	2014	3, 5, 7, 20 mo	3 + 1	Complicated pneumonia	Empyema	Cured	PCR	3	C
16	25/F	2015	2, 4, 6, 14 mo	3 + 1	Complicated pneumonia	Pleural effusion	Cured	Culture	14	C
17	29/M	2015	3, 5, 7, 18 mo	3 + 1	Complicated pneumonia	Necrotizing pneumonia	Cured	PCR	3	C
18	51/M	2015	2, 4, 6, 16 mo	3 + 1	Complicated pneumonia	Empyema	Cured	PCR	3	C
19	49/F	2015	12,14 mo	2	Complicated pneumonia	Empyema	Cured	PCR	3	C
20	54/F	2015	3, 5, 7,17 ​​mo	3 + 1	Complicated pneumonia	Empyema	Cured	PCR	3	C
21	58/M	2016	14, 16 mo	2	Complicated pneumonia	Empyema, necrotizing pneumonia	Pneumatocele	PCR	3	C
22	35/M	2016	2, 5, 9, 18 mo	3 + 1	Complicated pneumonia	Empyema, necrotizing pneumonia	Cured	PCR	3	C
23	41/M	2016	3, 4, 7, 16 mo	3 + 1	Complicated pneumonia	Necrotizing pneumonia	Cured	PCR	3	C
24	23/M	2016	3, 5, 6, 15 mo	3 + 1	Complicated pneumonia	Pleural effusion	Cured	PCR	3	C

*C, completed; DT, diagnostic technique; Pt, patient; Ser, serotype; UC, uncompleted. †Patient resident in Andorra (routine immunization schedule 2+1) and transferred to a participating hospital.

### Distribution of PCV13 Serotypes

Of the 104 cases of IPD caused by PCV13 serotypes, 23.1% (24 cases) were in age-appropriately vaccinated patients. The percentage of vaccine failure varied greatly according to the serotype: 38.1% (16/42) in serotype 3 cases, 29.4% (5/17) in serotype 19A cases, 7.7% (1/13) in serotype 14 cases, and 5.3% (1/19) in serotype 1 cases. Of the PCV13 serotypes, the proportion of serotype 3 in age-appropriately vaccinated patients was higher than in unvaccinated patients (66.7% vs. 27.8%; p = 0.001) (Table 2).

**Table 2:** Distribution of PCV13 serotypes causing invasive pneumococcal disease in patients 2–59 months of age in cases of vaccine failure and in unvaccinated patients, Catalonia, Spain, 2012–2016

PCV13 serotype	Vaccine failure, no. (%), n = 24	Unvaccinated patients, no. (%) n = 72	OR (95% CI)	p value
1	1 (4.2)	16 (22.2)	0.15 (0.02–1.21)	0.062
3	16 (66.7)	20 (27.8)	5.20 (1.93–14.04)	0.001
6A	0	1 (1.4)	0	0
6B	1 (4.2)	1 (1.4)	3.09 (0.19–51.35)	0.439
7F	0	3 (4.2)	0	0
9V	0	3 (4.2)	0	0
14	1 (4.2)	12 (16.7)	0.22 (0.03–1.77)	0.174
18C	0	1 (1.4)	0	0
19A	5 (20.8)	12 (16.7)	1.32 (0.41–4.21)	0.634
19F	0	2 (2.8)	0	0
23F	0	1 (1.4)	0	0

### Characteristics of IPD According to Vaccine Failure

We found no differences in sex or age group between cases of IPD with and without vaccine failure (Table 3). Cases with vaccine failure were associated with complicated pneumonia (OR 6.65, 95% CI 1.91–23.21). Pneumonia with empyema was the most common form of complicated pneumonia, both in cases of vaccine failure (71.4%) and in the remaining cases (77.5%). Cases of IPD with vaccine failure were associated with the diagnosis only by real-time PCR (OR 5.18, 95% CI 1.84–14.59). We observed no differences between the epidemiologic variables studied.

**Table 3:** Characteristics of cases of invasive pneumococcal disease in patients 2–59 months of age with and without vaccine failure, Catalonia, Spain, 2012–2016

Variable	Vaccine failure, no. (%), n = 24	No vaccine failure, no. (%), n = 156	OR (95% CI)	p value
Sex				
F	8 (33.3)	58 (37.2)	Referent	
M	16 (66.7)	98 (62.8)	1.18 (0.48–2.94)	0.716
Age group				
2–23 mo	9 (37.5)	73 (46.8)	Referent	
24–59 mo	15 (62.5)	83 (53.2)	1.46 (0.60–3.55)	0.395
Clinical form				
Meningitis	0	16 (10.3)	0	0
Septic shock	0	4 (2.6)	0	0
Uncomplicated pneumonia	1 (4.2)	26 (16.7)	0.22 (0.03–1.68)	0.134
Complicated pneumonia	21 (87.5)	80 (51.3)	6.65 (1.91 −23.21)	0,001
Occult bacteremia	0	20 (12.8)	0	0
Other	2 (8.3)*	10 (6.4)†	1.33 (0.27–6.46)	0.664
Pneumonia complication				
Empyema	15 (71.4)	62 (77.5)	0.73 (0.25–2.14)	0.561
Pleural effusion	3 (14.3)	23 (28.8)	0.41 (0.11–1.54)	0.263
Necrotizing pneumonia	8 (38.1)	18 (22.5)	2.12 (0.76–5.91)	0.146
Intensive care unit admission	2 (8.3)	35 (22.4)	0.31 (0.07–1.40)	0.173
Sequelae at discharge	3 (12.5)	20 (11.9)	0.96 (0.26–3.53)	1.000
Underlying disease	0	9 (5.8)	0	0
Diagnostic technique				
PCR only	19 (79.2)	66 (42.3)	6.62 (1.47–29.82)	0.014
Culture only	3 (12.5)	44 (28.2)	1.57 (0.25–9.84)	0.631
PCR + culture	2 (8.3)	46 (29.5)	Referent	
Breastfeeding	22 (91.7)	125 (81.2)	2.55 (0.57–11.47)	0.260
School or daycare attendance	20 (83.3)	102 (66.2)	2.55 (0.83–7.85)	0.104
Respiratory infection previous month	17 (70.8)	88 (57.1)	1.82 (0.71–4.65)	0.205
Recurrent otitis media	5 (20.8)	19 (12.3)	1.87 (0.62–5.59)	0.257

*Osteoarticular infection (1); mastoiditis (1).  †Osteoarticular infection (5); mastoiditis (4); orbital cellulitis (1).

### Characteristics of IPD Caused by Serotype 3 in Unvaccinated Cases and Cases with Vaccine Failure

Of the 42 case-patients with IPD caused by serotype 3, a total of 20 (47.6%) had received no dose of PCV13, 16 (38.1%) were age-appropriately vaccinated, and 6 (14.3%) were age-incorrectly vaccinated according to the SmPC. We found no significant differences between IPD caused by serotype 3 in unvaccinated patients and age-appropriately vaccinated patients with respect to the distribution by sex, age group, clinical form, and complications (Table 4). None of the severity factors compared (days of hospital admission, stay and days of admission to the ICU, complications, and sequelae at discharge) was associated with either group.

**Table 4:** Characteristics of cases of invasive pneumococcal disease caused by serotype 3 in patients 2–59 months of age with vaccine failure or unvaccinated, Catalonia, Spain, 2012–2016*

Variable	Vaccine failure, no. (%), n = 16	Unvaccinated, no. (%), n = 20	OR (95% CI)	p value
Sex				
F	5 (31.3)	6 (30.0)	Referent	
M	11 (68.8)	14 (70.0)	0.94 (0.23–3.92)	0.936
Days of admission, mean (SD)	14.69 (9.81)	12.25 (6.07)	1.04 (0.95–1.13)	0.367
ICU days, mean (SD)	1.00	3.00 (1.14)	Not calculable	0.540
Age group				
0–23 mo	3 (18.8)	7 (35.0)	Referent	
24–59 mo	13 (81.3)	13 (65.0)	2.33 (0.49–11.06)	0.456
Clinical form				
Septic shock	0	1 (5.0)	0	0
Uncomplicated pneumonia	0	2 (10.0)	0	0
Complicated pneumonia	15 (93.8)	17 (85.0)	2.65 (0.25–28.24)	0.613
Mastoiditis	1 (6.3)	0	0	0
Empyema				
No	3 (20.0)	3 (17.6)	Referent	
Yes	12 (80.0)	14 (82.4)	0.86 (0.15–5.06)	1.000
Pleural effusion				
No	14 (93.3)	13 (76.5)	Referent	
Yes	1 (6.7)	4 (23.5)	0.23 (0.02–2.36)	0.338
Necrotizing pneumonia				
No	9 (60.0)	13 (76.5)	Referent	
Yes	6 (40.0)	4 (23.5)	2.17 (0.47–9.95)	0.450
ICU				
No	15 (93.8)	18 (90.0)	Referent	
Yes	1 (6.3)	2 (10.0)	0.60 (0.05–7.28)	1.000
Sequelae at discharge				
No	13 (81.3)	17 (85.0)	Referent	
Yes	3 (18.8)	3 (15.0)	1.31 (0.23–7.57)	1.000
Underlying disease				
No	16 (100.0)	19 (95.0)		
Yes	0	1 (5.0)		
PCR diagnosis only				
No	1 (6.3)	2 (10.0)	Referent	
Yes	15 (93.8)	18 (90.0)	1.67 (0.14–20.23)	1.000

*ICU, intensive care unit.

## Discussion

Since the introduction of PCV13, several authors have reported cases of IPD caused by vaccine serotypes, both in patients who had received >1 dose of PCV13 and in age-appropriately vaccinated patients. In our study, cases with vaccine failure were associated mainly with serotype 3, the clinical presentation of complicated pneumonia, and patients without underlying conditions or other risk factors. This type of vaccine failure coincides with that found by Antachopoulos et al. in Greece (*19*) and confirms the results of Moraga-Llop et al. in Catalonia (*18*).

Kaplan et al. (*17*), in a study carried out in 8 US pediatric hospitals, found that vaccine failures were associated mainly with serotype 19A, age <6 months, and patients with underlying conditions. A retrospective study by Basaranoglu et al. (*27*) of IPD cases in children treated at a tertiary pediatric hospital in Ankara, Turkey, in 2015 reported 2 cases of vaccine failure associated with serotype 19F in patients with underlying neurologic disease. These studies differ from ours in 2 respects. First, they were carried out in a population for which pediatric PCV13 vaccination had been financed since 2010; thus, Basaranoglu et al. (*27*) found that the vaccination coverage in children was 96%. Our study was conducted in a population with an estimated vaccination coverage of 63% (*9*). In addition, although several studies indicate that there has been no decrease in the incidence of serotype 3 after the introduction of PCV13 (*9*,*28*,*29*), only the study in adults by Fukusumi et al. (*29*) included cases diagnosed by real-time PCR; therefore, it cannot be ruled out that, in a population with higher vaccination rates, there were fewer vaccine failures because of herd immunity. 

The second aspect is methodological. The studies by Kaplan et al. (*17*) and Basaranoglu et al. (*27*) included only cases diagnosed by culture. In our study, cases diagnosed by PCR were also included; in fact, cases of vaccine failure were associated with a diagnosis by PCR. Selva et al. (*30*) showed the importance of PCR in the diagnosis of IPD caused by serotype 3 in children <5 years of age with negative cultures. Almeida et al. (*31*), in a retrospective study conducted in patients <18 years of age admitted with pneumonia to a tertiary hospital in Portugal during 2012–2014 and diagnosed by PCR, found 4 cases of vaccine failure in patients who had received 4 doses of PCV13, which were all associated with serotype 3. Silva-Costa et al. (*32*) analyzed 152 pleural fluid samples from pediatric patients in Portugal during 2010–2015 to identify and serotype *S. pneumoniae*; 68% of cases were diagnosed only by PCR and, as in our study, the serotypes most frequently identified were 3, 1, and 19A. That study detected 19 cases of PCV13 vaccine failure, of which 17 were related to serotype 3. Serotype 3 was the most frequently identified serotype in children vaccinated with PCV13 in both studies and, although Silva-Costa et al. analyzed only cases of complicated pneumonia, a pathology associated with this serotype, our results, analyzing all clinical presentations of IPD, were identical. We also found a higher frequency of serotype 3 cases in patients with vaccine failure compared with unvaccinated patients, which is an indicator of the importance of this serotype in the post-PCV13 era. When the 2 studies were carried out, the vaccination coverage in Portugal (61%) was similar to that of Catalonia (63%).

As in the study by Kaplan et al. (*17*), we found that serotype 19A was associated with cases of PCV13 vaccine failure in children <2 years of age, the most frequently isolated serotype in this age group in our setting (*10*). Dominguez et al. (*9*) found that PCV13 effectiveness in the prevention of IPD caused by serotype 19A was 86% for >1 dose. The similar results observed by Kaplan et al. (*17*) for this serotype in a population with routine PCV13 vaccination may be explained by the fact that in half of the cases (5/10) reported by Kaplan et al., patients also had some type of underlying condition, whereas in our study, no patient had an underlying condition.

Heininger et al. (*26*) classified vaccine failures according to their cause or origin. Our study found no failures related to usage issues because, in all cases, the dates of vaccine administration and the number of doses administered according to the technical specifications were verified. The data in our study included cases during January 2012–June 2016, so the vaccines administered did not belong to the same batch. Although it is not known whether the preservation or storage of each vaccine was correct, given that vaccine failures occurred mostly in serotype 3 and in cases with complicated pneumonia, it seems unlikely that vaccine failures can be related to any of these factors, because in that case the case-mix of vaccine failures would be much more heterogeneous. Host-related vaccine failures also seem unlikely because no patient had a history of any underlying condition, including immunodeficiency. This finding is reinforced by the absence of differences between cases of IPD caused by serotype 3 in unvaccinated patients and in those with vaccine failure. It may be assumed that, if cases with vaccine failure had some type of immunodeficiency that would have induced a poor immunological response to the PCV13 vaccine, this effect would have manifested as a greater severity of IPD in cases with vaccine failure. The lack of significant differences in the epidemiologic variables between cases with and without vaccine failure makes it unlikely that vaccine failures were related to interference by other infectious agents. The fact that serotype 3 was associated with cases with vaccine failure, and specifically with age-appropriately vaccinated patients who had completed their vaccination schedule, indicates that vaccine failures could be vaccine related because of low PCV13 effectiveness with respect to serotype 3. This conclusion is in line with the results published by other authors (*9*,*13*,*33*) who studied PCV13 effectiveness in case-control studies and with the results reported on PCV13 effectiveness by Andrews et al. (*14*,*34*) in indirect cohort studies. All those authors observed that the effectiveness for all PCV13 serotypes was high, except for serotype 3, where it was very limited. Vanderkooi et al. (*11*) observed that the level of opsonophagocytic antibodies against serotype 3 was lower than that for the other PCV13 serotypes and Martinón-Torres et al. (*12*) observed that serotype 3 had the lowest levels of immunogenicity after PCV13 vaccination. Van der Linden et al. (*15*), looking at cases diagnosed only by culture, reported a PCV13 effectiveness against serotype 3 in children <24 months of age of 74% (95% CI 2%–93%) for those who had received >1 dose of vaccine but a nonsignificant effectiveness of 63% (95% CI −393% to 97%) for those who had received a postbooster dose.

A limitation of the study is that it was not possible to perform a serologic study to determine serotype-specific IgG concentrations. No specific studies were made to rule out immunodeficiency in cases with vaccine failure. However, the absence of a history of disease, the control of patients after discharge (which lasted 6 months in the case of discharge with sequelae), and the fact that IPD in cases with vaccine failure did not present greater severity than unvaccinated cases, support the validity of our results. Another limitation is that serotypes 25F and 38 were negative for the *wzg* (*cpsA*) gene. However, neither of these serotypes are PCV13 serotypes, and of the 89 strains serotyped using the Quellung reaction only 2 were serotype 38 and 1 serotype 25F.

In conclusion, after the introduction of the PCV13 vaccine in Spain, a significant number of IPD cases in Catalonia were recorded in the form of complicated pneumonia produced by PCV13 serotypes in age-appropriately vaccinated children without concurrent conditions. Serotype 3 represented 66.7% of cases with vaccine failure and was associated with age-appropriately vaccinated patients who had completed their vaccination schedule and with the 24–59 months age group. Serotype 19A represented 20.8% of cases of vaccine failure and was associated with age-appropriately vaccinated patients with an incomplete vaccination schedule and children <2 years of age. In 93.8% of vaccine failures associated with serotype 3, the diagnosis was made only by PCR, which suggests the importance of this diagnostic technique in avoiding underdetection of this serotype. Our findings indicate that vaccine coverage should be increased to reduce the circulation of vaccine serotypes. However, there are doubts as to whether PCV13 vaccination will reduce serotype 3 cases to the same extent as other vaccine serotypes. Continuous surveillance of cases of IPD using not only culture but also PCR to characterize vaccine failures is necessary.
